# Familial Carcinoma of the Duodenum in Adolescence

**DOI:** 10.1038/bjc.1949.38

**Published:** 1949-09

**Authors:** H. Ungar

## Abstract

**Images:**


### FAMILIAL CARCINOMA OF THE DUODENUM IN ADOLESCENCE.

H. UNGAR.

*From the Department of Pathology, Hadassah University Hospital, Jerusalem, Israel.*

Received for publication June 7, 1949.

CARCINOMA of the duodenum has been observed in this laboratory at autopsy in three young siblings whose case reports form the subject of this paper. The relation of these cases to generalized intestinal polyposis will be discussed.

### CASE I.

Rivkah A—, aged 19, was admitted to the Hadassah University Hospital in January, 1939.

*Previous history.*—The patient suffered from malaria and typhoid fever several years ago. In 1935 she experienced a sudden pain in her right eye; exophthalmos later developed on the same side. At examination in the Eye Clinic of the Hadassah Medical Organization " endothelioma " of the orbita was diagnosed. No biopsy was made.

*Recent history.*—Two months prior to admission to the hospital, patient complained of attacks of pain in the upper part of the abdomen. A blood count revealed considerable secondary anaemia. During 5 weeks prior to admission progressive jaundice was present.

Two days before admission the temperature rose to 40° C., and a chill was experienced.

During the fortnight between admission and death there was intermittent fever, the temperature ranging between 38° and 40° C. During the same period, the jaundice subsided notably. Abdominal pain attacks occurred with increasing frequency and intensity. There were repeated attacks of chills; blood culture revealed *Staphylococcus aureus.* Terminally, bronchopneumonia developed. The final clinical diagnosis was as follows: Septicaemia. Obstructive jaundice, possibly due to pressure of enlarged lymph nodes. Systemic disease of the lymphatic system, ? Hodgkin's disease.

### *Autopsy Findings.*

*Anatomical diagnosis.*

Adenocarcinoma and papilliferous carcinoma of the duodenum (surrounding the papilla of Vater); hepatic abscesses due to ascending infection of bile ducts. Papillary malignant adenoma of duodenum. Multiple polyps of colon. Extra-bulbar tumour of right orbita (clinical diagnosis " endothelioma "). Cavernous haemangioma of the base of tongue.

*Record of anatomical findings (abbreviated).*

Autopsy was performed 3 hours after death by evisceration through a small incision below the right costal arch. The pertinent findings were as follows:

*Duodenum* (Fig. 1).—In the area of the papilla of Vater, there was a wide defect with a depressed centre whose floor was torn and rugged. The defect measured 3-4 cm. in diameter. The elevated edges were in part undermined, showing papilliform lobulations. The entire area was adherent to the pancreas. In sections through the centre of the ulceration the layers of the duodenal wall were found obliterated but scarcely thickened. Between the duodenum and pancreas a layer of firm whitish tissue was present.

Separated from the tumour by a small segment of normal mucosa, there was a pedunculated node about the size of a walnut, the surface of which exhibited papillary formations. Within the same segment of the intestine, a second pedunculated pea-sized node of similar appearance was found.

In the second and third portion of the duodenum numerous small formations which appeared in some areas as small polyps and in other areas as tiny ridges were found (Fig. 4). These formations measured between 2 and 4 mm. in diameter. They were localized either between the plicae or at their height. They were found either singly or crowded together.

*Jejuno-ileum.*—Without pathological changes.

*Colon.*—The mucosa of the transverse colon contained two pea-sized polyps which were rather firm and could be shifted with ease on the underlying tissues.

*Liver.*—Weight 2090 g. Marked enlargement. Cross-sections revealed in the upper part of the right lobe several cavities in irregular distribution which measured 1 to 4 cm. in diameter and contained a thick yellowish pus. The walls of the cavities were lined by a membrane-like yellowish layer, about 1 mm. in width. The surrounding liver tissue was a yellowish-brown colour. The acini were distinctly marked.

*Gall bladder.*—Without changes. Bile ducts throughout their whole extent without changes.

### *Histological Examination.*

1. *Sections from the ulcerated tumour.*—Within wide necrotic areas there were islets of columnar-cell carcinoma which in some areas assumed the character of adenocarcinoma. The occasionally papillary architecture was suggestive of a tumour which could have originated in a papillary adenoma (Fig. 5). In other areas the carcinoma infiltrated into the muscular layers in solid strands but did not invade the pancreas. Epithelium presented marked pleomorphism. The stroma was sparsely developed ; it was infiltrated by scattered small round cells.

2. *Small polypoid formations in the duodenum* were examined in several blocks from different areas. The examination revealed several histological aspects which may be regarded as representative of successive stages in the development of polyps.

In the area of the prominence, the submucosa was widened and the muscularis mucosae showed a funnel-shaped protrusion in the direction of the intestinal lumen. The whole formation was covered by a mucous membrane which was not different from the surrounding mucosa. Other polyps were chiefly formed by hypertrophic mucosa and only near the base was the submucosa slightly thickened.

3. *Fragments from the two largest papillary polyps in the duodenum* (Fig. 6).—These polyps were built upon an axis of loose connective tissue, fat tissue and a muscularis mucosae which ramified into papillae. The glands were crowded together ; their ramified lumina showed variations in width and tortuosity ; frequently papillary projections into the lumen were seen. Groups of condensed glandular formations often assumed a nodular form and in some instances were outlined by a thin layer of smooth musculature. In some places the glandular epithelia were piled up ; they did not reach beyond the base of the glands. The nuclei were dark-staining and columnar. The cytoplasm was scanty and dark-oxyphilic. Scattered mitotic figures were present. Toward the base of the papilloma, the epithelium assumed a more quiet character and gradually goblet cells became more numerous. In the continuation, there were Brunner's glands without abnormalities.

The irregular hyperplasia of the glands, the lack of goblet cells, the dark-staining nuclei and the appearance of typical and atypical mitotic figures justify the diagnosis of an early malignant change.

4. *Polyp of colon* (Fig. 7) was entirely composed of mucosal glands which were irregularly wide. Their epithelium, like that of adjacent mucosa, was without abnormalities. The surface of the polyp was ulcerated and there was superficial granulation tissue.

### CASE II.

Chajim A—, aged 16 at the time of death. The earlier history contained no contributory information. Fourteen months prior to admission he first began to complain of bouts of cramp in the abdomen. These occurred at irregular intervals and were without clear localization.

The patient was admitted to the hospital in June, 1939, three months before his death. At admission his chief complaints were shortness of breath, attacks of tachycardia, lack of appetite and abdominal colic localized under the costal arch on either side. During the preceding year the patient had lost a great deal of weight. There were recurrent periods of diarrhoea. Marked secondary anaemia was found. Examination of stools showed ascaris and taenia ; occult blood was repeatedly present. Frank blood was seen in stools only a short time before death. X-ray examination of stomach and duodenum failed to reveal pathological changes.

The attacks of abdominal pain became more frequent and more intense during the patient's stay in the hospital. The patient died about three months after admission.

### *Autopsy Findings.*

The autopsy was done 18 hours post mortem by a short abdominal incision. Signs of decomposition were then already in evidence.

*Anatomical diagnosis.*

Columnar-cell carcinoma of duodenum with partial change to carcinoma simplex, necrosis, ulceration and continuous invasion of regional and adjacent mesenteric lymph glands. Papillary adenoma of Lieberkuhns glands of duodenum. Multiple polyps of duodenum.

*Record of anatomical findings* (abbreviated).

*Abdominal cavity.*—Liver extended by 4 cm. below the right costal margin. Peritoneum was smooth and glistening. Small intestines were distended by splashing contents. Caecum and colon showed considerable meteorism. Intestinal wall exhibited dark-red and greenish discolouration due to post-mortem changes.

*Duodenum* (Fig. 2).—At a distance of about 1·5 cm. behind the pylorus there was a walnut-sized polyp on a short and narrow pedicle. The duodenal mucosa as far as 1 cm. behind the papilla of Vater was without lesions. Choledochus and pancreatic duct without abnormalities.

Beyond the papilla of Vater, the lumen was considerably distended. There was a nearly circular, crateriform defect, which extended from the posterior wall, including almost the entire circumference of the duodenum. The lesion, which measured 7 to 8 cm. in diameter, showed a rugged floor ; it was entirely composed of tumour tissue and blended underneath with enlarged lymphatic glands of the peripancreatic group. The latter were glued together and had been completely replaced by tumour. Sections through the floor of the ulceration revealed a firm and, in some places, a friable tissue which was of a pale greyish-yellowish colour. The tumour was defined against the head of the pancreas by a thin layer of greyish-red connective tissue.

The remaining portion of the duodenum was considerably distended and filled with greenish fluid. The mucosa was extremely pale ; it exhibited several soft tiny polypoid or ridge-like elevations which could be moved with the mucosa. They were of a uniform size and only rarely as high as the plicae transversae.

Because of the advanced state of the post mortem changes, close examination of the remainder of the intestines was not attempted.

*Abdominal lymph nodes.*—The lumps of peripancreatic nodes which have been mentioned were completely fused with lymph nodes in the upper portion of the mesenteric root and around the adjacent portion of the inferior vena cava, both of which had been overrun by tumour tissue. Other mesenteric glands were free of pathological lesions.

### *Histological Examination.*

1. *Sections from the large ulcerated tumour* revealed a carcinoma which grew in some areas in solid strands, while in others gland-like formations predominated. Epithelia showed a considerable degree of pleomorphism and there were numerous typical and atypical mitotic figures. Near the margin of the ulcerated portion, the tumour was seen to extend into muscular and submucosal layers.

2. *Pedunculated tumour between pylorus and papilla of Vater.*—This presented formations of a papilloma which was built around ramifications of submucosa and muscularis mucosae. The main mass of the node was made up of glandular tissue with regular lumina which in places were slightly tortuous. Epithelium was columnar with dark nuclei and oxyphilic cytoplasm. There was no pleomorphism or mitotic activity. No goblet cells were present.

Between the glands there was a fibrous stroma which in most areas was sparsely developed. It contained infiltrations of small round cells, plasma cells and scattered foci of polymorphonuclear leucocytes. Brunner's glands were without pathological lesions. In some places single dark-celled glands projected from the head of the papilloma between the acini of Brunner's glands.

3. *Duodenal wall with small polyps.*—The histological aspects of the small polyps closely resembled those observed in Case I.

### Case III.

Joseph A—, aged 18, was admitted for the first time to the Hadassah University Hospital in August, 1944, with the diagnosis of acute appendicitis followed by perforation.

Previous history not contributory.

On operation, a fluctuating mass, which was believed to be a perityphlitic abscess, was found above the ileocaecal region. Appendix was not removed.

The wound was drained and healed within a month by secondary intention. Following laparotomy the patient lost rapidly in strength, and concomitantly a gradual increase in the size of the abdomen was noted. Temperature remained normal ; bowel movements were regular.

Three months after the operation, the patient was re-admitted to the Hospital because of severe intestinal haemorrhage. A firm huge tumour mass was palpable in the right half of the abdomen extending from the costal arch to the height of the crista iliaca. Having regard to the familial history a probable diagnosis of duodenal carcinoma was made. Patient received repeated blood transfusions. He died nine days following admission.

#### *Autopsy Findings.*

*Anatomical diagnosis.*

Adenocarcinoma of the terminal portion of duodenum.

Metastatic carcinoma in parapancreatic and paraportal lymph nodes.

Chronic encapsulated peritoneal abscess resulting from two fistulae, on the floor of two ulcers (non-neoplastic) in the upper jejunum.

Acute generalized suppurative peritonitis due to perforation of the encapsulated peritoneal abscess.

Polyposis of small intestines ; single polyps of colon.

Extreme anaemia of tissues ; septic hyperplasia of spleen.

Terminal endocarditis of tricuspid and mitral valve.

*Summary of autopsy record* ($1\frac{1}{2}$ hours after death).

Body extremely emaciated. Pallor of skin and visible mucous membranes. Abdomen markedly distended and tense.

*Abdominal cavity.*—Entire peritoneum was covered by fibrinous deposits There were easily severable adhesions between intestines and the anterior abdominal wall, mostly on the right side. Only in the right upper quadrant were the adhesions firm and included a cavity, about the size of the grape-fruit, which was defined by the anterior wall of the duodenum, the transverse colon near the hepatic flexure, a small portion of the descending colon and several loops of the jejunum. The cavity, which contained a dark feculant fluid, connected with the rest of the peritoneal sac by a small rent in the direction of the caecum. Two fistular openings in the wall of the jejunum established connection between the intestinal lumen and the described " cavity."

*Duodenum.*—The first and second portions of the duodenum were moderately distended. Between the plicae, the mucosa exhibited numerous raised nodules and polyps which near their tip measured about 2–3 mm. in diameter (Fig. 8).

From a point about 20 cm. below the pylorus and extending to the jejunal flexure a huge tumour mass had transformed the duodenum into a wide sac (Fig. 3). In this area the internal surface of the duodenum was made rugged by vegetations of friable tissue which presented a moist and greyish cut-surface, frequently including yellowish specks or wider areas of the same colour. Tissue of this character extended through all layers of the intestinal wall. In some cases the tissues were broken down and only a paper-thin membrane separated the lumen from adjacent structures.

The posterior wall of the duodenum was adherent to nodular masses which were formed as described above by parapancreatic and paraportal lymph nodes being replaced by tumour tissue.

*Jejuno-ileum*—Mucosa was extremely pale. There were tiny polyps of the kind seen in the first and second portions of the duodenum. In the direction of the ileum the number of polyps became less, and in the ileum they were sparse.

About 20 cm. behind the large tumour, there were two ulcerations, each measuring about 10 cm. in diameter. Within their limit the intestinal wall showed firm thickening, and the floor contained narrow fistules which opened into the abscess cavity.

*Colon.*—Mucosa was pale ; it presented a few flat elevations which measured between 3 and 5 cm. in diameter. Two small polyps were found near the hepatic flexure and in the transverse colon. The heads of the polyp measured about 4 mm. in diameter and were slightly redder in colour and of the same consistency as the surrounding mucosa.

### *Histological Examination.*

*Duodenum.*—Medullary carcinoma containing only very sparse stroma. The cells exhibited extreme pleomorphism and typical and atypical mitotic figures were found in abundance. Extensive necrosis was present in many areas.

*Jejunum.*—In several fragments taken for examination, small polyps were seen, some of them arising from the side of jejunal folds, others lying between the latter. The histological aspects were in no way different from those found in Cases I and II.

*Colon.*—The pedicle of the polyps was built of submucosa and muscularis mucosae. The mucosa near the tip of the polyps was slightly thicker than in their vicinity. The glands displayed slight irregularities in outline and some of them were moderately dilated. In several glands the goblet cells were replaced by groups of dark epithelia containing eosinophilic cytoplasm. In the vicinity of one of the polyps small foci of thickening of the mucous membrane due to increase of glandular tissue were found.

---

### EXPLANATION OF PLATES.

FIG. 1.—Duodenum with pylorus in Case I. Besides the ulcerated carcinoma, there are several adenomatous polyps (Fig. 6) and in the upper third of the photograph, numerous small polyps (Fig. 4).

FIG. 2.—Case II : Between the large ulcerated carcinoma and pylorus a pedunculated polyp is seen and distally from the carcinoma there are scattered polyps.

FIG. 3.—Case III : Large ulcerated carcinoma. The floor of the ulcer is formed by lumps of lymph-nodes with metastatic carcinoma. Between carcinoma and pylorus there are innumerable small polyps (Fig. 8).

FIG. 4.—Polyposis of duodenum in Case I.

FIG. 5.—Papillary carcinoma (Case I).

FIG. 6.—Adenomatous polyp of duodenum (Case I).

FIG. 7.—Adenomatous polyp of colon (Case I).

FIG. 8.—Polyposis of duodenum in Case III.

1

2

3

4

Ungar.

5

6

7

8

Ungar.

### FAMILY HISTORY OF CASES SEEN AT AUTOPSY.

The parents, of Yemenite Jewish stock, were born in the Old City Quarter of Jerusalem and there spent all their life. No previous illnesses were remembered. They married in 1918 and had nine children (Table I). Five children died. Three of them—the first, the third and the fourth—were examined post mortem. The findings in these form the subject of this article. Of the two others, it is known that one died from " toxicosis " in infancy and one from typhoid fever. In these cases, no autopsy was performed.

TABLE I.

Yaacob ♂ — Sarah ♀

| Rivkah | Yehuda | Chaim | Itzhak | Eliezer | Rachel | Simha | Moshe | Joseph |
|---|---|---|---|---|---|---|---|---|
| ♀ | ♂ | ♂ | ♂ | ♂ | ♀ | ♂ | ♂ | ♂ |
| b. 1919, d. 1939. Carcinoma duodeni. | b. 1921. | b. 1923, d. 1939. Carcinoma duodeni. | b. 1926, d. 1944. Carcinoma duodeni. | b. 1928. | b. 1929, d. 1929. " Toxicosis in infancy." | b. 1931, d. 1939. Typhoid fever. | b. 1934. | b. 1936. |

The following is known concerning the four surviving children :

The now eldest son Yehuda was born in 1921. His previous history contains no data of pertinent interest. In 1944 he underwent an operation for a traumatic lesion of the liver. During laparotomy the operating surgeon, who was aware of our interest in the case, palpated the intestines but found no evidence of a tumour. An X-ray examination of the stomach and duodenum 2 months following the operation likewise failed to reveal pathologic changes.

The three younger boys were kept under observation from birth by the Infant Welfare Centres and the School Hygiene Departments of the Hadassah Medical Organisation. The respective records contain no references to gastro-intestinal disorders.

In 1946 two X-ray examinations of the 20 years, old boy Eliezer were performed. No abnormalities were disclosed by the radiographs.

### COMMENT.

A search of the literature failed to reveal any previous report on appearance of duodenal tumour in several members of one family. Only nine cases of duodenal carcinoma during the first and second decade of life could be collected (Table II).

TABLE II.—*Carcinoma Duodeni in Patients below the Age of* 20.

| Author. | Year. | Age of patient. |
|---|---|---|
| Ewald* | 1896 | 16 years |
| Bernouilli* | .. | 18 ,, |
| Whittier† | 1889 | Case 1 : 17 years |
| | | ,, 2 : 18 ,, |
| | | ,, 3 : 18 ,, |
| Brill | 1904 | 17 years |
| Oberndorfer | 1929 | 13 ,, |
| Lieber, Stewart and Lund | 1939 | 15 ,, |
| Hoffman and Pack (Case 11) | 1937 | 18 ,, |

* Quoted from Borrman (1926).
† Quoted from Hoffman and Pack (1937).

There are, however, in the literature several cases in which epithelial tumour in the duodenum was co-existing with multiple benign or malignant tumour in other parts of the gastro-intestinal tract (Table III).

TABLE III.—*Cases in which Epithelial Tumour of the Duodenum Co-existed with Epithelial Tumours in other Portions of the Gastro-intestinal Tract.*

| Author. | Age and sex of patient. | Localization of: Benign polyposis. | Malignant tumours. |
|---|---|---|---|
| 1. Hauser (1895)* | M. 33 years | Duodenal polyp, polyposis recti et colon | Carcinoma colon. |
| 2. Petrow (1896)† | F. 20 " | Duodenum, jejunum, ileum | .. |
| 3. Funkenstein (1904)* | F. 24 " | Duodenum, stomach, colon, rectum | .. |
| 4. Döhring (1907) | F. 32 " | Duodenum, stomach, caecum, colon | Four carcinomas in transverse colon and rectum. |
| 5. Versé (1908)* | F. 32 " | Adenomas in duodenum, multiple polyps in caecum, colon, rectum | .. |
| 6. Wechselmann (1910) (case 1) | F. 62 " | Duodenum, jejunum, colon | .. |
| 7. Maurice (1913) (quoted after Wirth) | F. 23 " | Polyps in duodenum and jejunum | .. |
| 8. Nagel (1925) | ? | Duodenum, jejunum | Malignant adenoma of duodenum, carcinoma ventriculi. |
| 9. Markus (1933) | F. 40 years | Benign polyposis of stomach | Malignant adenoma of duodenum. |
| 10. Cassidy and Maschia (1934) | F. 29 " | Polyps in duodenum and jejunum | Several malignant polyps. |

* Quoted from Golden.

† Quoted from Döhring.

The comparison of these cases and of our own with generalized polyposis of the intestines is of interest. In this condition benign polyps exhibit a definite tendency to malignant change. The disease is hereditary and affects patients of all age groups, including the lowest. Since generalized polyposis is known to involve predominantly the large intestine, it is not infrequently mentioned in textbooks and articles under the name " polyposis or adenomatosis of the colon." However, in rare cases, the disease was found to be prevalent in the small intestine. Besides the instances mentioned in Table III, others of this variant have been described by Lubarsch in 1888 (Döhring 1907), Schwytzer (1924), Roan (1932), Haggard and Floyd (1935), Ravitch (1948).

Some pathologists have taken the view that generalized polyposis is an entity which since it causes characteristic severe dysentery-like symptoms and malignant change in a high percentage of cases, should be separated from that represented by cases in which only few polyps are formed (Oberndoerfer, 1929; Lockhart-Mummery, 1934; Hullsiek, 1928). On this view our Case III and probably also Case I would certainly have to be comprehended under generalized polyposis, but Case II, which presented only scattered small polyps besides the carcinoma of the duodenum, would not belong to this group.

Recent experience has tended, however, to detract from the significance of the nosologic separation of " generalized polyposis " from " single or multiple polyps." Several authors were able to show that lesions of this type need not be congenital and can arise at various times over a protracted period of life (McKenney, 1936 ; Lockhart-Mummery and Dukes, 1928 ; Haggard and Floyd, 1935). It has become evident that differences in the speed of progress of the disease can delay its clinical manifestation even to the seventh and eighth decade of life (Kaufmann, 1931 ; Coffey and Bargen, 1939). Considering the high incidence of malignant change in intestinal polyposis, an estimated 62 per cent to almost 100 per cent of the cases (Lockhart-Mummery and Dukes, 1928 ; Coffey and Bargen, 1939), it is to be expected that in certain cases patients may die from intestinal carcinoma before the polyposis has had time to generalize. This possibility is evident also from genealogical tables of the affected families, in which cases of generalized polyposis, single polyps and carcinoma of the intestines without any polyps occur randomly (Jüngling, 1928).

It may be inferred from the foregoing that the hereditary and familial character of the disease is the decisive factor in the diagnosis. The designation of all three of our cases as generalized polyposis therefore seems to be justified.

The observations reported in this article are relevant to the understanding of factors which govern the manifestation of malignancy in intestinal polyposis. Several authors have assumed a casual relationship between the appearance of polyps and carcinoma in any given case. They believed that the chance for malignant transformation is increased when there is a numerical increase of polyps (Lockhart-Mummery, 1934 ; Hurst, 1939). Other investigators have related the sites of predilection of intestinal carcinoma to the greater irritation of the mucosa in the areas of physiological faecal stasis, viz. the rectum, sigmoid colon, the region of the colic flexures and near the ileo-caecal valves (Oberndoerfer, 1929).

On the other hand, the published histories of families in which malignant neoplasm appeared in several members favour the assumption that the site of malignant manifestation is determined by one specific genetic agent and that the histological character and also the age of onset of the malignant change are determined independently (Macklin, 1935, 1938). The action of a genetic agent for the site of the malignant transformation in previously benign polyposis cannot be proved beyond doubt in a localization, e.g. rectum or colon, in which the incidence of intestinal carcinoma is high even in non-hereditary cases. However, in view of the extreme rarity of the development of carcinoma of the duodenum in the earlier decades of life, the operation in our cases of all three mentioned factors appears to be very probable.

### SUMMARY.

Three instances of carcinoma of the duodenum with concomitant polyposis of the small intestine and single polyps of the colon are reported.

The patients were siblings, two brothers and a sister, 16, 18 and 19 years of age respectively.

The literature on duodenal carcinoma in young patients is reviewed. Possible implications of this observation for the understanding of malignant transformation in intestinal polyposis are discussed.

Permission to use the clinical records was kindly given by Dr. M. Rachmilewitz and Professor J. Kleeberg, heads of the Departments of Medicine of the Hadassah University Hospital in Jerusalem.

### REFERENCES.

BORRMANN, R.—(1926) ' Handb. Spez. Pathol. Anat.', Vol. IV, pt. 1. Berlin.
BRILL, N. E.—(1904) *Amer. J. med. Sci.*, **128**, 824.
CASSIDY, J. M., AND MACCHIA, B. J.—(1934) *Amer. J. digest. Dis.*, **1**, 755.
COFFEY, R. J., AND BARGEN, J. A.—(1939) *Surg. Gynec. Obstet.*, **69**, 136.
DÖHRING, H.—(1907) *Arch. klin. Chir.*, **83**, 194.
GOLDEN, R.—(1928) *Amer. J. Roentgenol.*, **20**, 405.
HAGGARD, W. D., AND FLOYD, W. O.—(1935) *Amer. J. Surg.*, **31**, 428.
HOFFMAN, W. J., AND PACK, G. T.—(1937) *Arch. Surg.*, **35**, 11.
HULLSIEK, H. E.—(1928) *Surg. Gynec. Obstet.*, **47**, 346.
HURST, A.—(1939) *Lancet*, i, 553, 621.
JÜNGLING, O.—(1928) *Beitr. klin. Chir.*, **143**, 476.
KAUFMANN, E.—(1931) ' Lehrbuch der Speziellen Pathologischen Anatomie,' 9th ed. Berlin : de Gruyter.
LIEBER, M. M., STEWART, H. L., AND LUND, H.—(1939) *Ann. Surg.*, **109**, 383.
LOCKHART-MUMMERY, J. P.—(1934) *Ibid.*, **99**, 178.
*Idem*, AND DUKES, C.—(1928) *Surg. Gynec. Obstet.*, **46**, 591.
MACKLIN, M. T.—(1935) *Medicine*, **14**, 1.—(1938) Univ. Wisconsin, Symposium on Cancer, p. 32.
MCKENNEY, D. C.—(1936) *J. Amer. med. Ass.*, **107**, 1871.
MARKUS, H.—(1933) *Klin. Wschr.*, **12**, 617.
NAGEL, G. W.—(1925) *Arch. Surg.*, **11**, 529.
OBERNDOERFER, S.—(1929) ' Handb. Spez. Pathol. Anat.', Vol. IV, pt. 3. Berlin.
RAVITCH, M. M.—(1948) *Ann. Surg.*, **128**, 283.
ROAN, O.—(1932) *Tex. St. J. Med.*, **27**, 782.
SCHWYTZER, R.—(1924) *Schweiz. med. Wschr.*, **5**, 869.
WECHSELMANN, L.—(1910) *Beitr. klin. Chir.*, **70**, 855.
WIRTH, W.—(1913) Inaug.-Dissertation, Berlin.


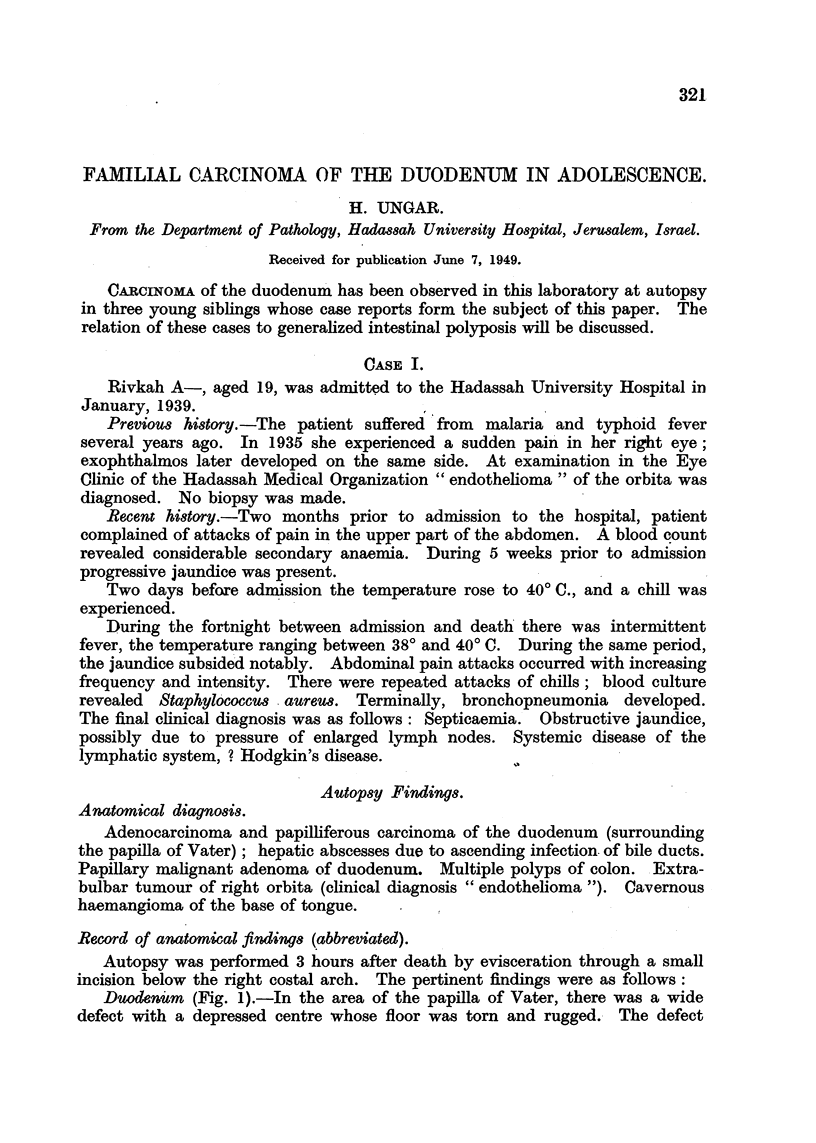

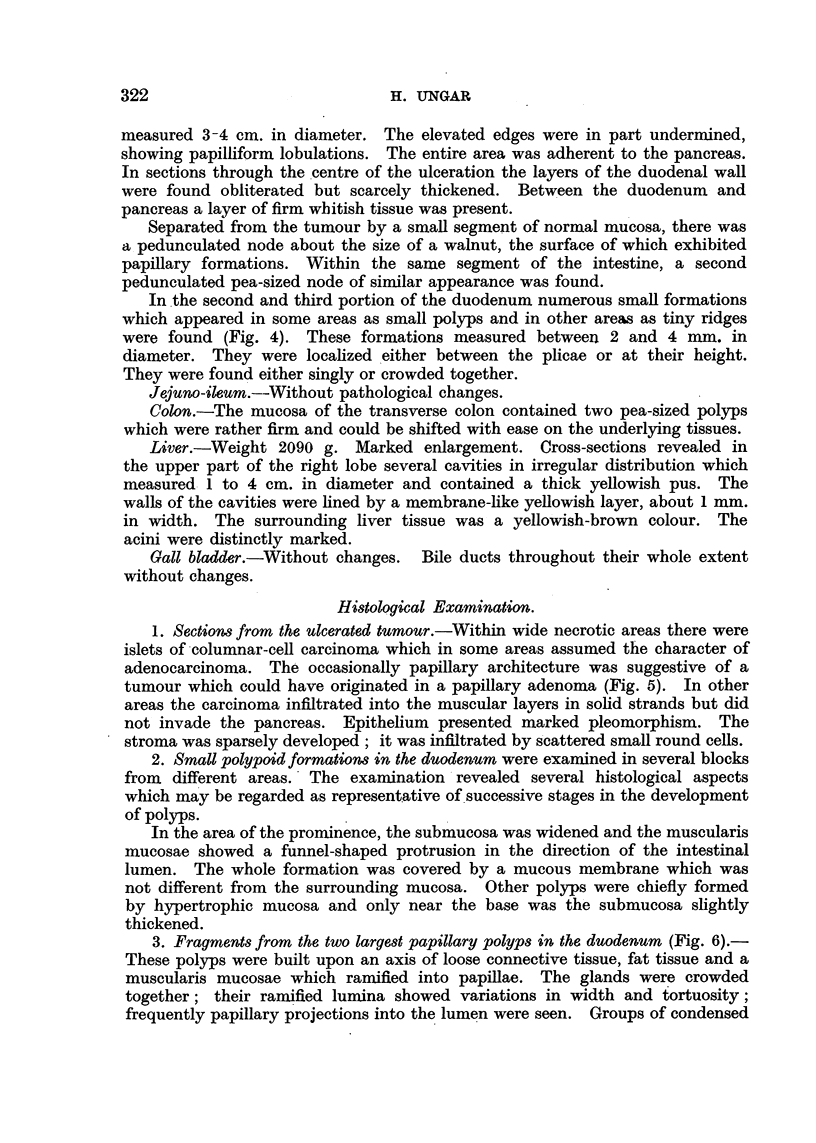

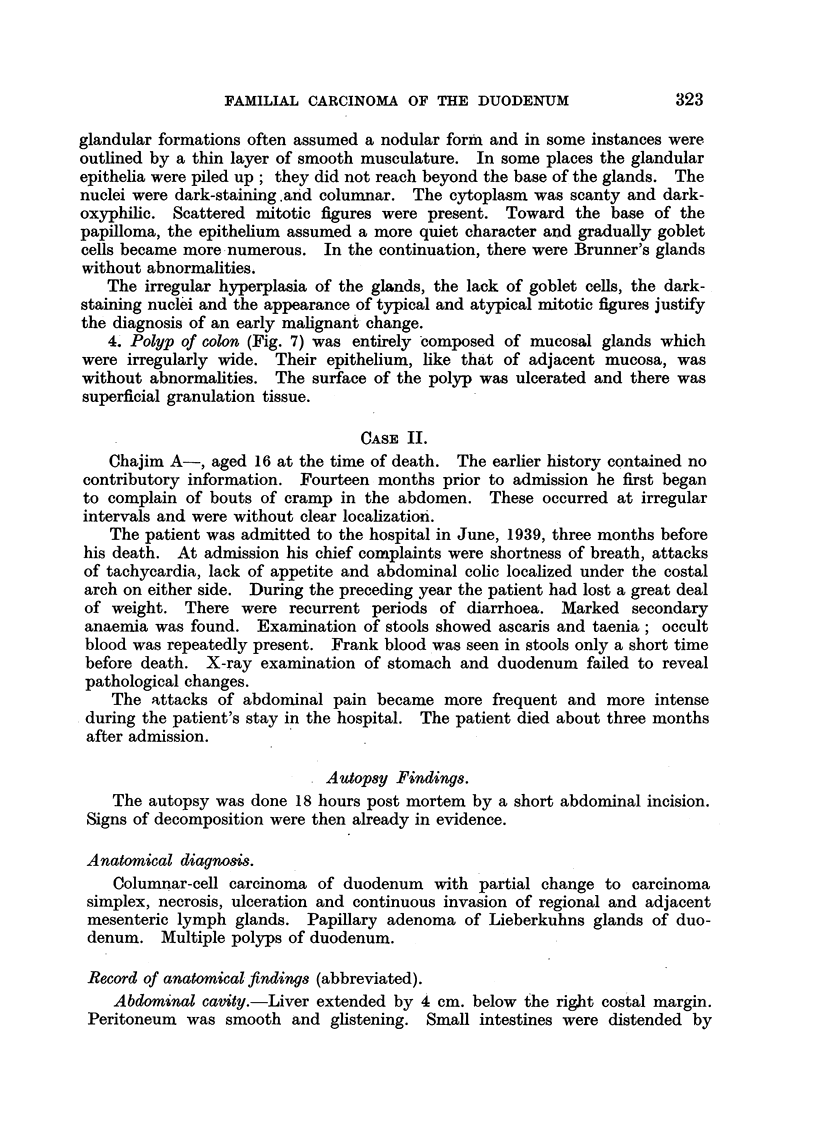

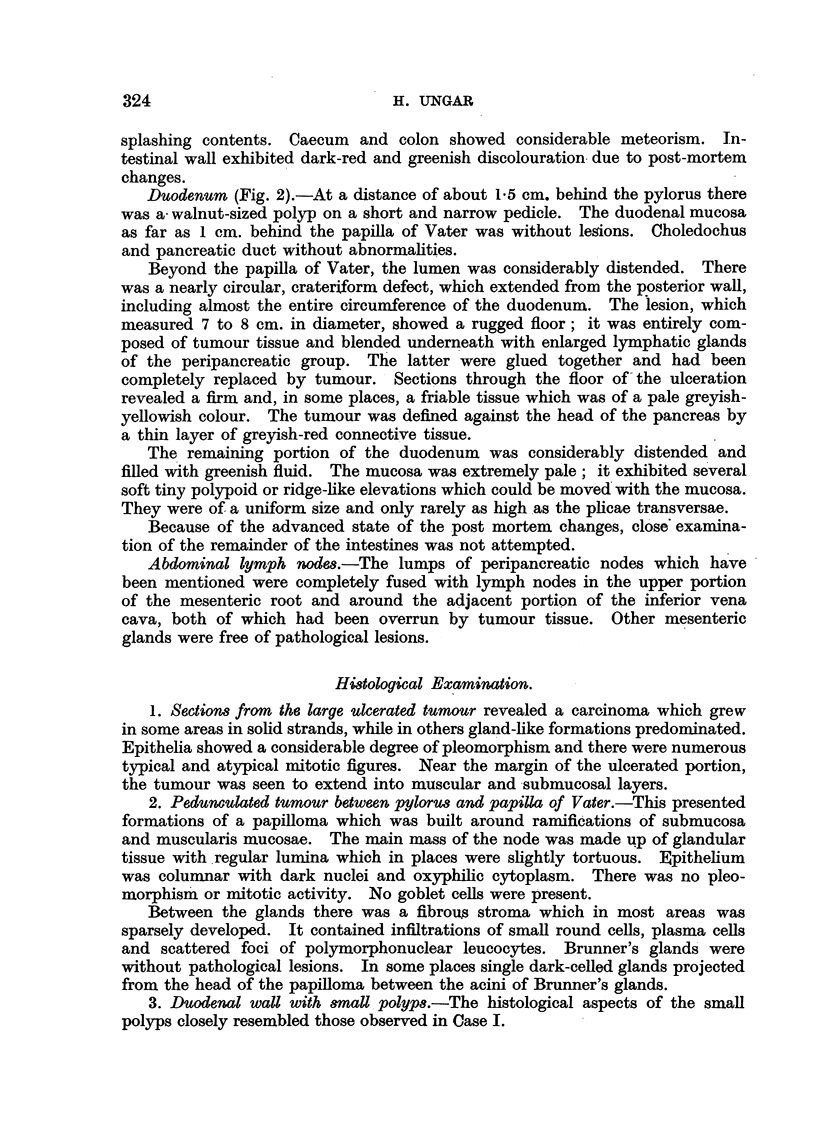

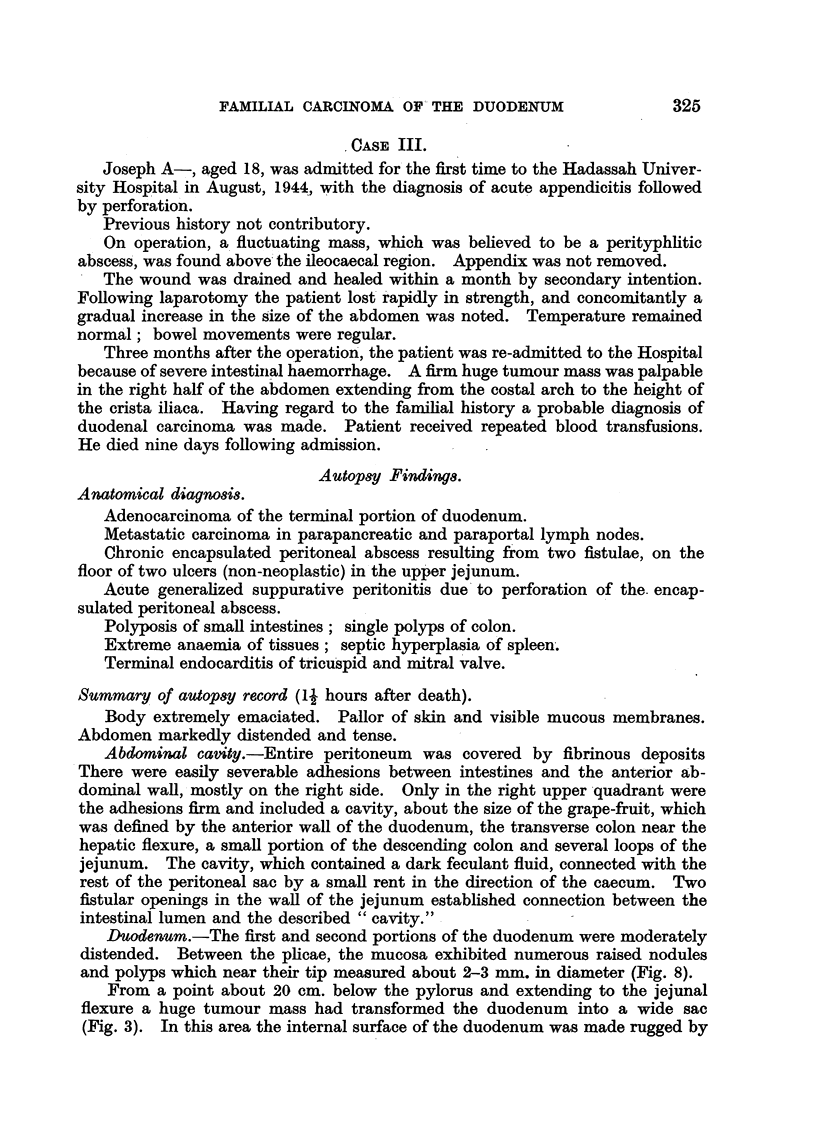

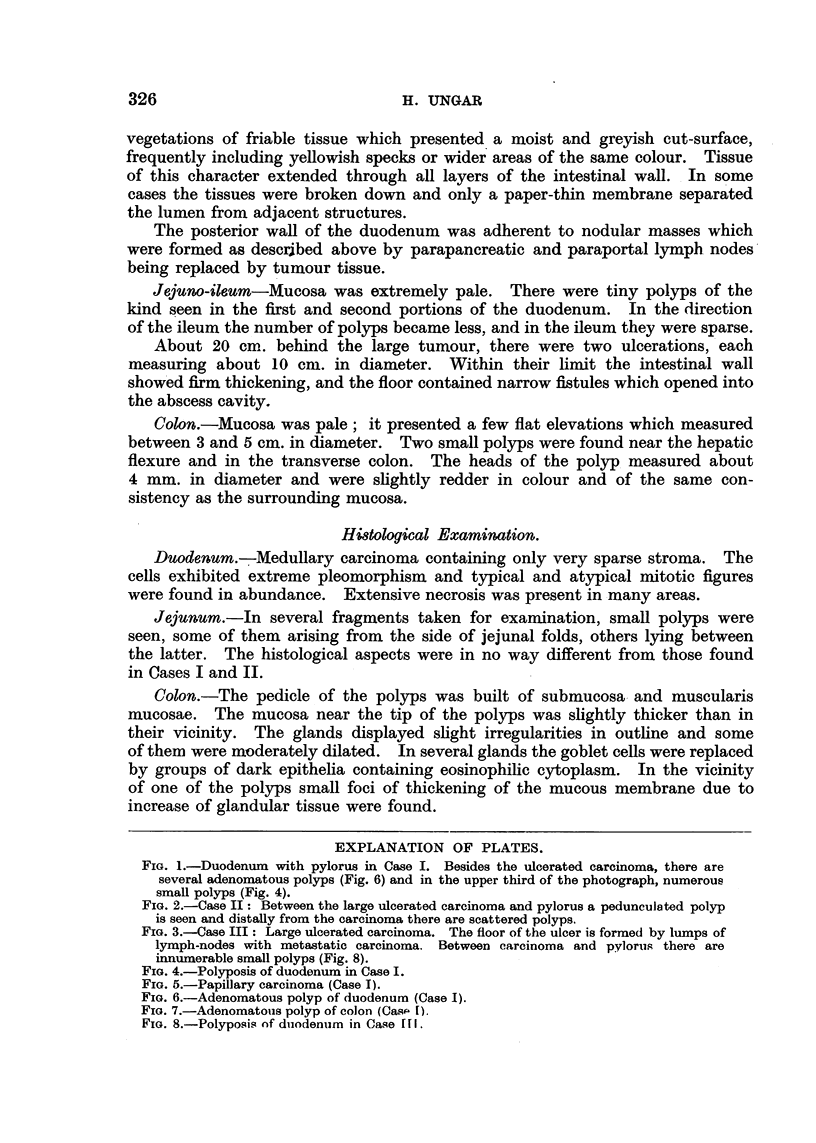

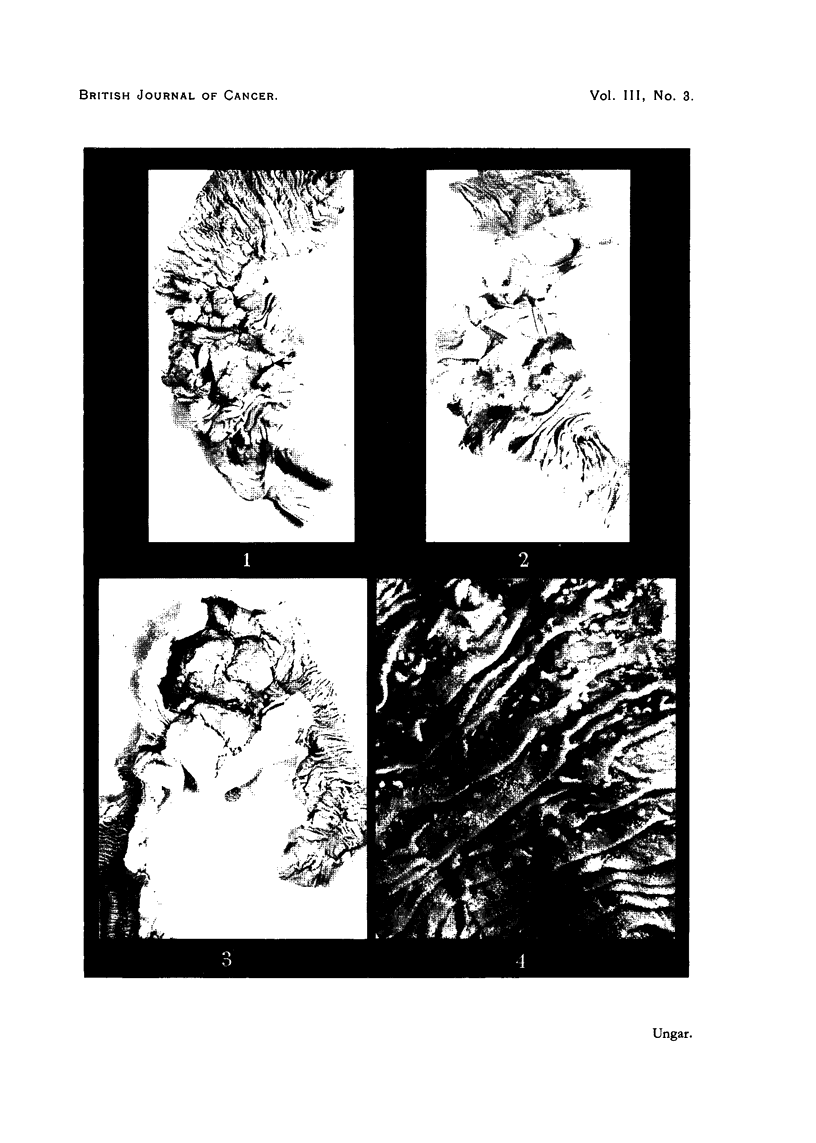

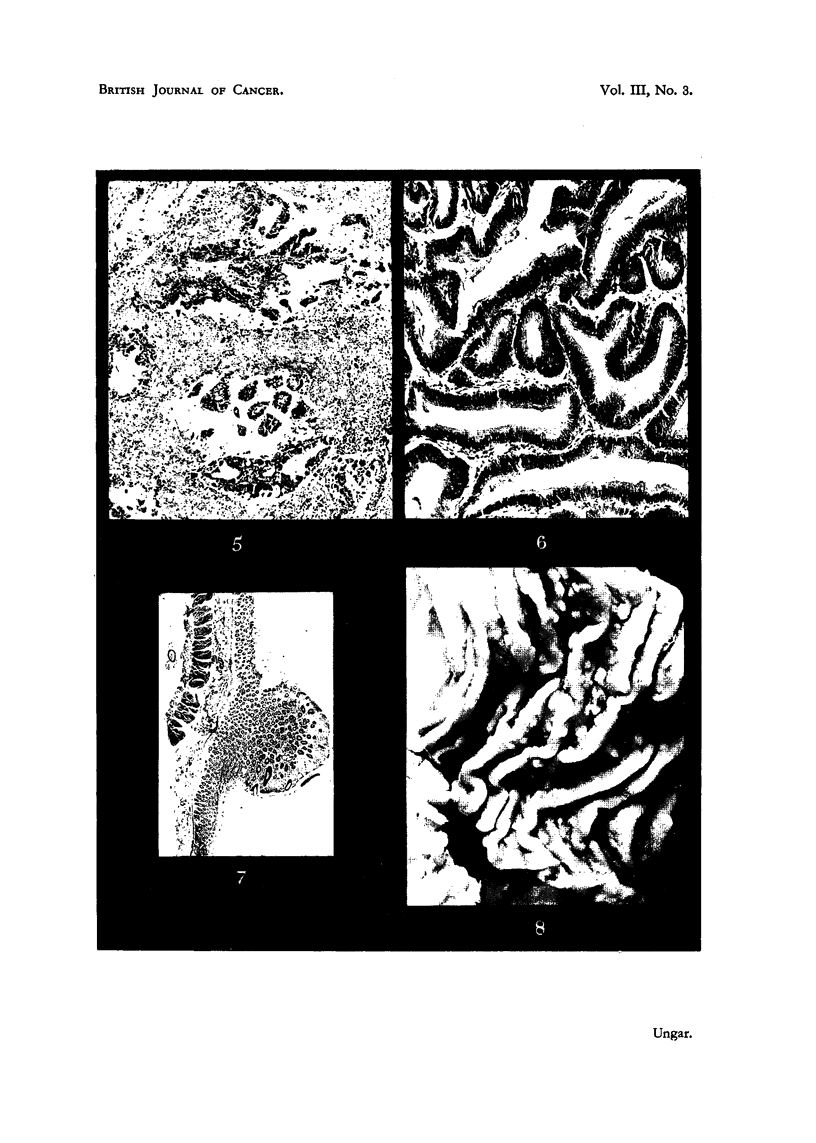

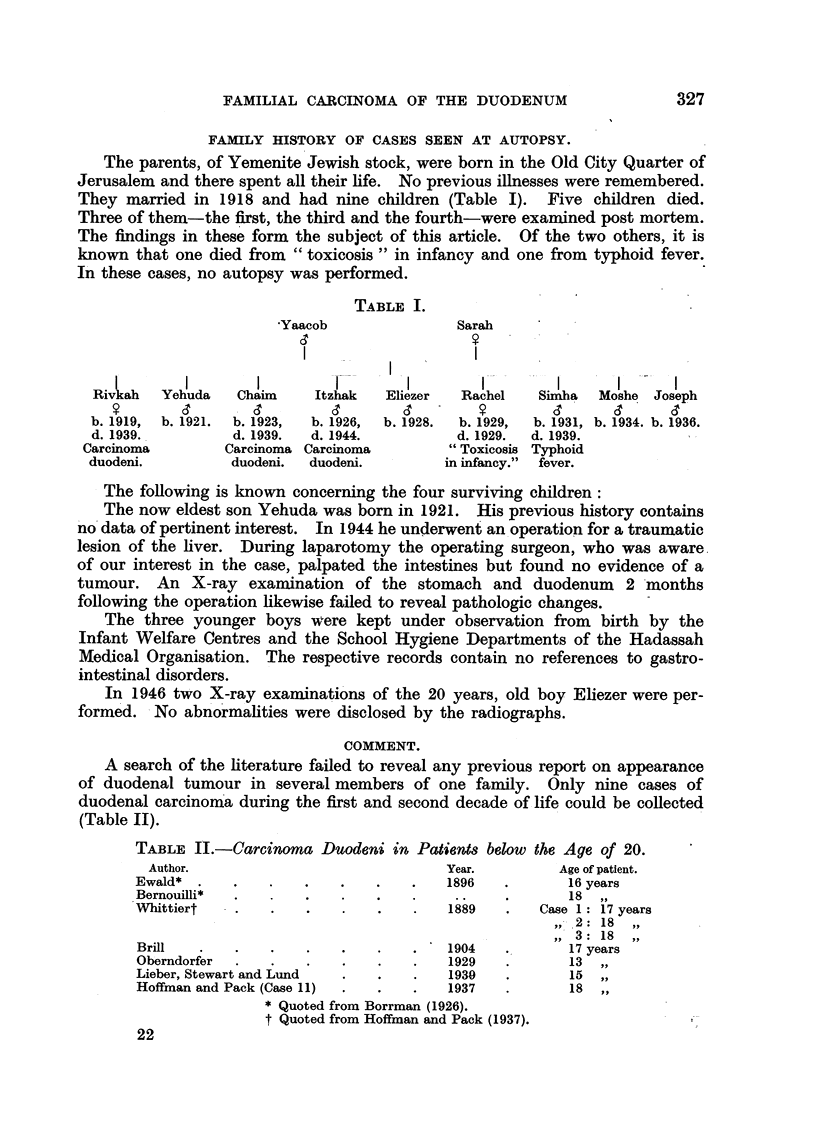

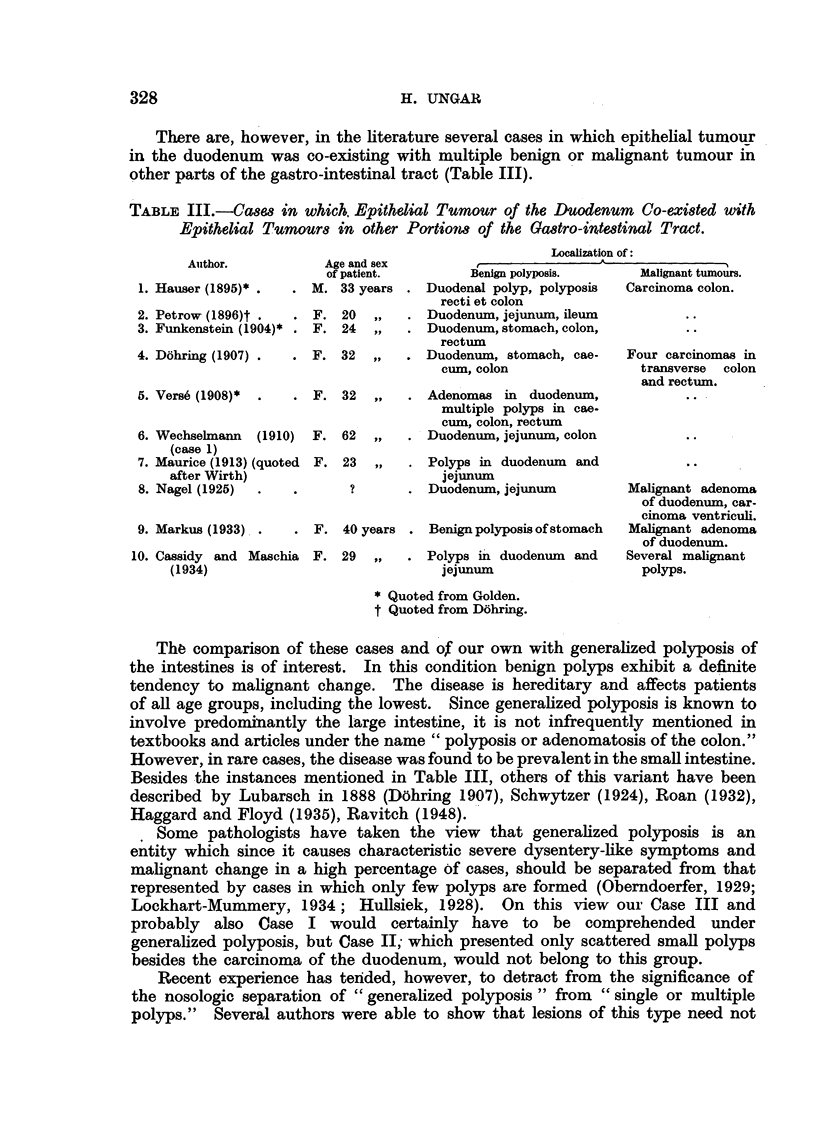

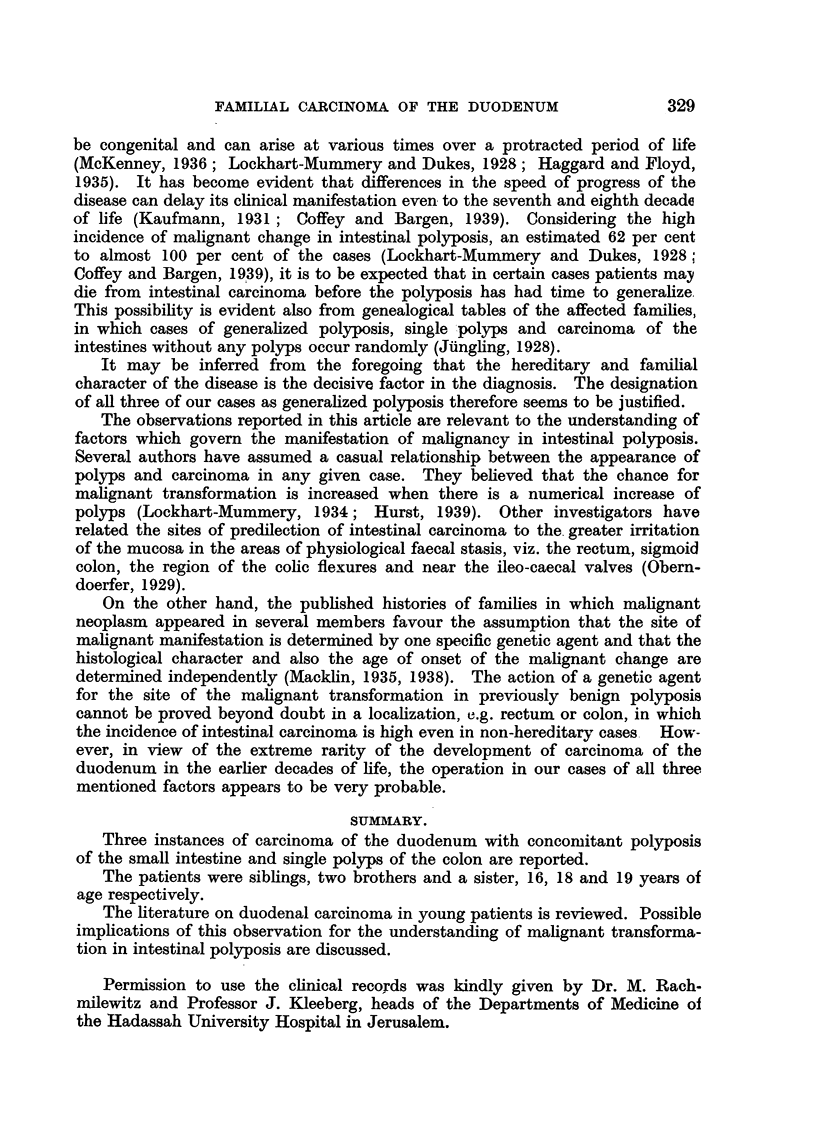

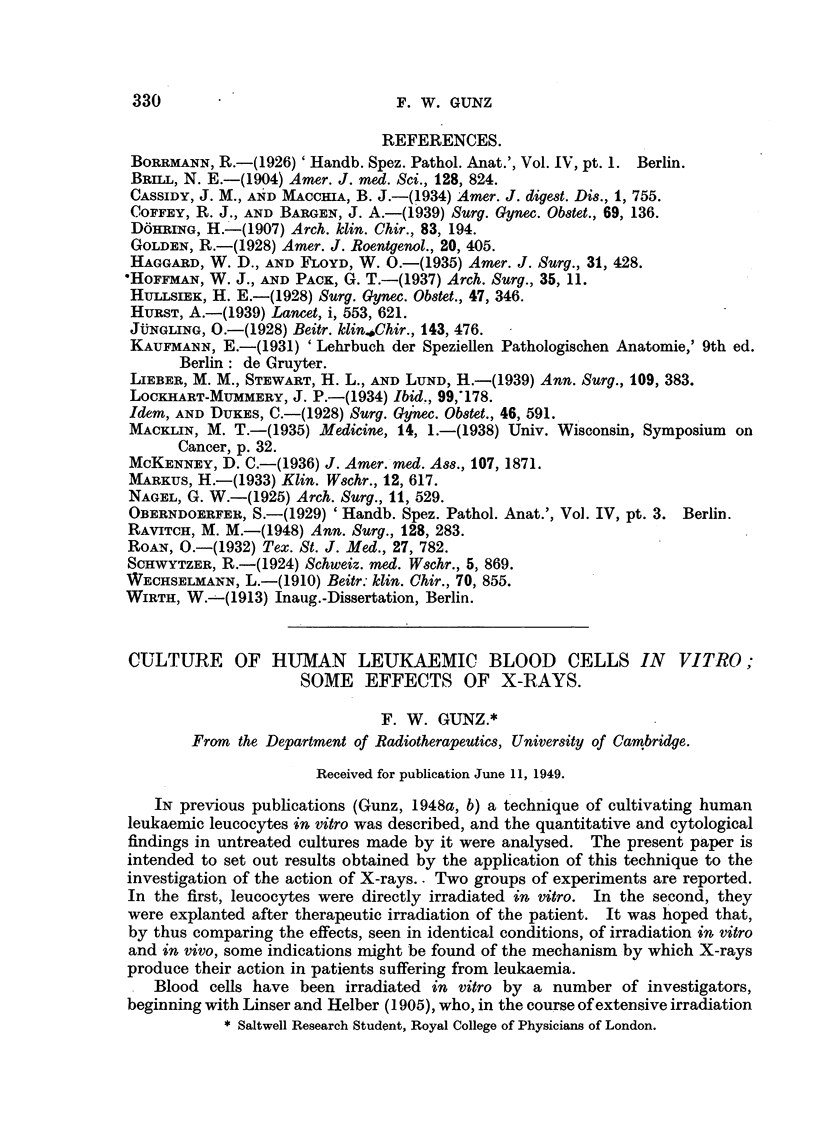


## References

[OCR_00735] Lockhart-Mummery J. P. (1934). THE CAUSATION AND TREATMENT OF MULTIPLE ADENOMATOSIS OF THE COLON.. Ann Surg.

[OCR_00747] Ravitch M. M. (1948). Polypoid Adenomatosis of the Entire Gastrointestinal Tract.. Ann Surg.

